# The effect of graded exposure therapy in patients with non-specific chronic lower back pain and kinesiophobia in Gauteng, South Africa

**DOI:** 10.4102/sajp.v82i1.2333

**Published:** 2026-07-08

**Authors:** Sandy Lord, Vaneshveri Naidoo, Jasmina Govind

**Affiliations:** 1 Department of Physiotherapy, Faculty of Health Sciences, University of the Witwatersrand, Johannesburg, South Africa

**Keywords:** chronic pain, kinesiophobia, graded exposure therapy, non-specific chronic back pain, fear avoidance, physiotherapy

## Abstract

**Background:**

Kinesiophobia hampers positive outcomes in patients with non-specific chronic lower back pain (NSCLBP). Graded exposure therapy is a psychosocial approach aimed at increasing patients’ functional capacity by challenging their fears and negative expectations.

**Objectives:**

Our study aimed to evaluate the effect of a graded exposure therapy programme on patients with NSCLBP, with primary outcomes assessing changes in pain intensity (Brief Pain Inventory – Short Form [BPI-SF]) and kinesiophobia (Tampa Scale of Kinesiophobia-11 [TSK-11]) from baseline to post-intervention and secondary outcomes assessing changes in pain interference (BPI-SF).

**Method:**

A quasi-experimental pre-test and post-test design was used. Twenty-eight participants with NSCLBP for more than 3 months and a TSK-11 score ≥ 22 were recruited. Outcome measures included the TSK-11, BPI-SF and Fear of Daily Activities Questionnaire (FDAQ). The intervention included a 4-week graded exposure programme and pain neuroscience education in the first session only. Data were analysed using the parametric paired *t*-test and Cohen’s *d*.

**Results:**

Out of 28 participants, most were female (96.4%) with a mean age of 45 (± 8.2) years and reported pain for more than 12 months. Significant improvements were observed on both the TSK-11 and BPI-SF (*p* < 0.001). Large effect sizes were observed for both kinesiophobia and pain severity (TSK-11: *d* = 1.73, 95% confidence interval [CI 1.14, 2.32]; BPI Pain Severity: *d* = 1.21, 95% CI [0.72, 1.70]).

**Conclusion:**

Graded exposure therapy was effective in reducing pain and kinesiophobia in patients with NSCLBP in the short term.

**Clinical implications:**

Future considerations should be given to implementing the graded exposure therapy in conjunction with other physiotherapy modalities.

## Introduction

The International Association for the Study of Pain (IASP) defined pain as ‘an unpleasant sensory and emotional experience associated with, or resembling that associated with, actual or potential tissue damage’ (Raja et al. 2020:14). This definition indicates that pain is a personal experience made up of multiple circumstances that may influence an individual’s well-being (Raja et al. 2020). Chronic pain is defined as ‘pain in one or more anatomical regions that persists or recurs for longer than 3 months, is associated with significant emotional distress, and/or significant functional disability’ (Nicholas et al. 2019:30). Chronic pain affects at least 10% of the world’s population annually (Jackson, Stabile & McQueen 2014). In South Africa, the prevalence of chronic pain is 18.3%, with an incidence of 30.5% of those cases having pain specific to their back (Kamerman et al. 2020). This high prevalence and incidence may be due to lower back pain (LBP) being a low-priority condition within the healthcare system, with poor implementation of strategies and policies addressing the management of LBP (Morris et al. 2018).

Non-specific chronic lower back pain (NSCLBP) negatively impacts quality of life and causes disability, affecting recovery and the ability to function normally (López-de-Uraide-Villanueva et al. 2015). This is due to factors like reduced mobility, loss of independence, emotional distress, impaired social and work function, pain catastrophising and kinesiophobia (Miller et al. 2020; Roditi & Robinson 2011). Increased disability and fear avoidance further diminish quality of life and complicate work and social activities (Comachio et al. 2018; Guclu et al. 2012; Stefane et al. 2013). Kinesiophobia is an excessive, irrational fear of movement and activity due to perceived vulnerability to pain or re-injury (Vlaeyen et al. 2004). This fear leads to reduced function, movement and dependence, further decreasing quality of life (Miller et al. 2020).

Graded exposure therapy is a cognitive process used to address kinesiophobia associated with chronic pain, by exposing patients to their fearful activities to improve their functional ability and reduce their pain-related fear (Leeuw et al. 2008; Roditi & Robinson 2011; Schemer et al. 2018; Jensen 2011). This is different to graded activity, which uses quota-based exercises that focus on the patient’s functional ability and are gradually increased irrespective of the patient’s pain levels (Kuss et al. 2016). Systematic reviews conducted by López-de-Uraide-Villanueva et al. (2015) and Macedo et al. (2010) have reported that graded exposure was more favourable compared to graded activity for the management of patients with NSCLBP, as it directly targeted the patient’s fears related to an activity more than their functional limitations.

Almost one in five people in South Africa are living with chronic pain (Kamerman et al. 2020) and a study conducted in sub-Saharan Africa reported that 70.8% of patients with chronic lower back pain (CLBP) present with kinesiophobia (Tiaho et al. 2021). Patients with NSCLBP and associated kinesiophobia are often seen for an extended amount of time by the physiotherapist, increasing the healthcare utilisation in South Africa and often with no improvement in their pain or outcomes (Kahere et al. 2022). Conventional physiotherapy, such as exercise and manual therapy, is effective in treating the physical limitations of NSCLBP; however, a gap is evident: psychosocial factors such as kinesiophobia are not addressed by physiotherapists (Garland & Jones 2019; Magalhães et al. 2018). Current literature describes the different strategies in the management of NSCLBP, such as graded activity, graded exposure therapy, therapeutic exercises, Pilates and pain neuroscience education (PNE) (Alonso-Sal et al. 2024; Roditi & Robinson 2011; Van Middelkoop et al. 2010). In South Africa, research is limited on the effects of graded exposure therapy in the management of NSCLBP (Gatchel et al. 2016; George & Zeppieri 2009). Our study aimed to determine the effect of graded exposure therapy in the management of patients with NSCLBP and associated kinesiophobia. The study tested the alternative hypothesis that graded exposure therapy would lead to reductions in pain intensity and kinesiophobia. The primary outcomes were changes in pain intensity (Brief Pain Inventory – Short Form [BPI-SF]) and kinesiophobia (Tampa Scale of Kinesiophobia-11 [TSK-11]), with pain interference (BPI-SF) assessed as a secondary outcome.

## Research methods and design

### Study design and setting

This was a quasi-experimental study, using a pre-test and post-test design. The reporting of our study adheres to the Transparent Reporting of Evaluations with Nonrandomised Designs (TREND) guidelines. Participants were recruited from January 2021 to August 2021 during the coronavirus disease 2019 (COVID-19) pandemic from a public district hospital physiotherapy outpatient department and within the various departments in the hospital in Gauteng. Adults 18 years or older with NSCLBP for more than 3 months and moderate to high levels of kinesiophobia indicated by TSK-11 scores of 22 and above were included in the study (Hapidou et al. 2012). Patients with back pain caused by spinal fractures or cancer, neurological signs and symptoms, and low levels of kinesiophobia indicated by TSK-11 scores below 22 or awaiting spinal surgery were excluded from the study.

### Study sample

The sample size for our study consisted of 28 participants. This was established using the number of patients with back pain (*n* = 172) seen in the physiotherapy department within 6 months, an estimated chronic pain prevalence of 18.3% derived from a study by Kamerman et al. (2020) reflecting a comparable demographic and the estimated 71.7% prevalence of kinesiophobia among individuals with CLBP derived from a study by Odole et al. (2016) with similar characteristics using the TSK. An effective sample size was calculated using the estimated number of patients with chronic back pain and kinesiophobia and the StatCalc Calculator with a margin of error set at 5% and an effect value of 1.0. Participants from the pilot study, which followed the same protocol with identical procedures and outcome measures, were included in the main study dataset to maximise statistical power and avoid unnecessary data loss, as no changes were made to the data collection procedures after the pilot study.

### Outcome measures

Three outcome measures were used pre-intervention and post-intervention. The TSK-11 is a self-report measure developed to assess the fear of movement in patients with chronic pain (Chimenti et al. 2021). Multiple studies report a high test-retest reliability (Intraclass Correlation Coefficient [ICC] = 0.81–0.93) and a high internal consistency (α = 0.68–0.80) of the TSK-11 in patients with CLBP (Calley et al. 2010; Chapman et al. 2011; George et al. 2010; Hapidou et al. 2012). The BPI-SF is a nine-item self-report measure widely used to assess pain intensity and the impact of pain on functional ability. The BPI-SF is a valid and reliable outcome measure with a good internal consistency of α = 0.85 for the pain severity subscale and α = 0.88 for the pain interference subscale and a high test and retest reliability (Mendoza et al. 2006; Tan et al. 2004). A licence agreement to use the BPI-SF was obtained from the University of Texas, M.D. Anderson Cancer Centre. The Fear of Daily Activities Questionnaire (FDAQ) is a self-report measure used in a physiotherapy setting. It is aimed at determining graded exposure therapy treatment plans and monitoring changes in fear levels. The FDAQ is a valid and reliable outcome measure with an internal consistency (α = 0.91) and a good test-retest reliability (Gay et al. 2015; George et al. 2009). The data were collected and stored electronically on a computer in a document that was password-protected pre-intervention and post-intervention. All participants were assigned a code by the research assistant (RA) that was used for data management and analysis.

### Procedure

The primary investigator (PI) and the RA in our study were both physiotherapists. The RA collected demographic data and administered the outcome measures (TSK-11, BPI-SF and FDAQ) and captured the data on an Excel spreadsheet. The PI conducted the intervention, which included an education session and graded exposure therapy. The intervention was tailored to each participant using the principles of graded exposure therapy to provide better person-centred care.

Ethics clearance was obtained from the University of the Witwatersrand Human Research Ethics Committee (Clearance Certificate No. M200733). The participants received detailed information sheets and informed consent forms by the RA. Once informed consent was obtained, baseline demographic information, BPI-SF and FDAQ were collected by the RA. The PI was blinded to the baseline data. At the start of the first treatment, a 30 min PNE session was conducted using a lecture with visual aids and an interactive discussion (Appendix 1). The key concepts of the PNE were to define chronic pain and kinesiophobia, to explain the fear avoidance model and neurophysiology of pain and to discuss the awareness of the participants on pain being an experience that can be managed (Louw et al. 2016; Vieira & Pimenta 2016).

The graded exposure therapy followed the PNE. The participants feared activities from the FDAQ were ranked from the least feared to most feared (López-de-Uraide-Villanueva et al. 2015; Macedo et al. 2010). The participant’s two most feared activities were used in the treatment sessions (George et al. 2010; George & Zeppieri 2009; Nicholas & George 2011; Schemer et al. 2019). For example, if the participant was fearful of reaching the floor, the participant was instructed to try to reach the floor. This was repeated until they reached a level that did not cause an increase in fear to determine a baseline activity level. The activity would be done with the participant attempting to reach the floor the number of times they could manage, then the activity progressed. If there were more than two highly rated feared, the participant chose the activity they needed to perform daily at work or at home. For the FDAQ activities requiring participants to carry objects weighing above or below 9.1 kg, 5-L plastic bottles filled with sand and equipped with handles were used. The bottles were weighed at different weights ranging from 1 kg to 10 kg.

The participants’ expectations of fear and pain were recorded pre-exposure and post-exposure of an activity using a monitoring tool (Appendix 2) with scores rated on a numerical pain rating scale like the FDAQ. The progression of the activity was determined by a reduction of fear by 10 points or more post-activity that was maintained at the next session. Progression was achieved by increasing the duration, frequency or intensity of the activity by at least 10% of the current level (George et al. 2008; Nicholas & George 2011). The type of progression was determined by the activity type and the participant’s limiting factor. For example, if the feared activity was climbing stairs and the participant was only able to climb up five stairs, the activity would be progressed by increasing the number of stairs climbed to six or seven stairs, which was determined by the participant and how many more they could do before feeling the fear rise again. If fear levels were increased or maintained, the activity was continued as per the previous session (George et al. 2010; George & Zeppieri 2009; Vlaeyen et al. 2002). The goal was for the participant to continue exposing themselves to their feared activities once the previous activity level was no longer feared. Therefore, as a home programme, the participant was encouraged to continue the activities at the same level that was covered during the treatment session, daily for the week until the next treatment session. Goals were set in consultation with the participant, based on work or home requirements or on how many repetitions of the feared activity they could do without an increase in fear. Magalhães et al. (2018) recommended this basis of goal setting, with the progression being the increase of the intensity of the activity according to the participants’ functional goals. A distress protocol had been developed for participants who had experienced increased anxiety or panic attacks; however, this was not used as none of the participants experienced these symptoms. The first treatment session for each participant was an hour and 15 min long. The follow-up treatment sessions were 30 min each and included the graded exposure therapy only. Sessions were once a week for a period of four weeks. The number of sessions were benchmarked from multiple studies using a similar treatment protocol and inclusion criteria and used an average of four treatment sessions (George & Zeppieri 2009; Vieira & Pimenta 2016; Woods & Asmundson 2008). Post-intervention data (BPI-SF and FDAQ) were collected and captured by the RA.

### Data analysis

The PI was blinded to the pre-test results of the TSK-11 and BPI-SF, as all outcome measures were administered and captured by the RA. Once data collection was complete and collated by the RA, the anonymised pre-test and post-test results were analysed by the PI under the analytical and methodological guidance of a statistician from the University of Witwatersrand, using Statistica version 14.0.0. The statistician was blinded to the timepoints to minimise analytical bias. The parametric paired *t*-test was used with a significance level set at *p* < 0.05. Effect sizes were calculated using Cohen’s *d*, with values around 0.2 and less interpreted as small, between 0.2 and 0.5 as medium, and 0.8 or greater as large (Aarts, Van den Akker & Winkens 2014). Per protocol analysis was used in our study.

### Ethical considerations

Ethical clearance to conduct our study was obtained from the University of the Witwatersrand Human Research Ethics Committee (No. M200733).

## Results

**FIGURE 1:**
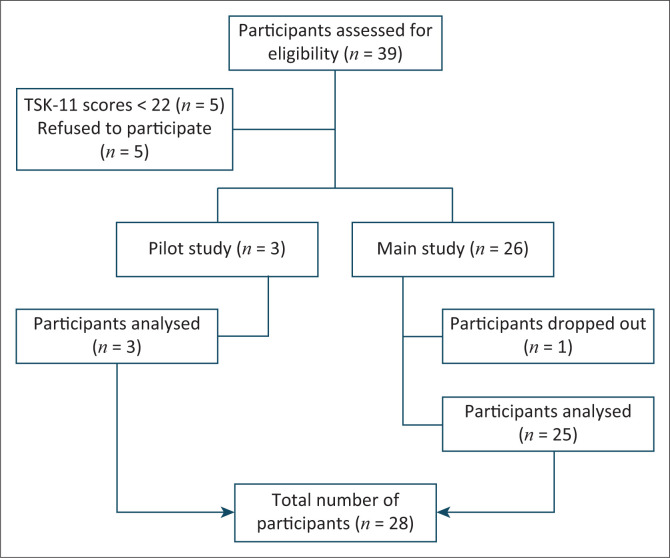
Summary of study sample.

The study sample is shown in Figure 1. One participant dropped out from the main study due to testing positive for COVID-19. The demographic data of the study participants are presented in Table 1. Most of the participants were females with ages ranging from 29 years to 59 years. The distribution of the duration of pain is presented in Table 2.

**TABLE 1:** Summary of demographic data of participants (*N* = 28).

Characteristic	Value	*n*	%
**Age (years)**			
Mean ± s.d.	45 ± 8.2	-	-
Range	29 – 59	-	-
**Gender**			
Female	-	27	96.4
Male	-	1	3.6

s.d., standard deviation.

**TABLE 2:** Summary of duration of pain (*N* = 28).

Duration of pain (months)	Frequency (*n*)
3–6	7
7–9	6
10–12	5
> 12	10

### Kinesiophobia

**FIGURE 2:**
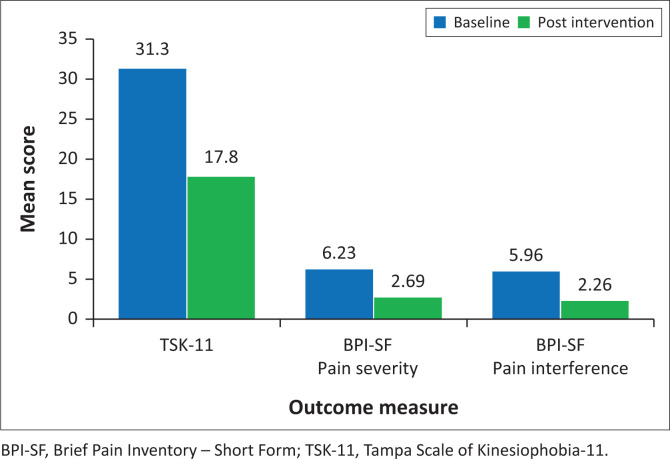
The mean scores at baseline and post-intervention for each outcome measure.

The data obtained from the TSK-11 at baseline and post-intervention were normally distributed. The results from the parametric paired *t*-test and Cohen’s *d* are presented in Table 3. Figure 2 shows a comparison between mean TSK-11 values at baseline and post-intervention, demonstrating a clinically significant reduction in kinesiophobia. The parametric paired *t*-test indicated a statistically significant difference (*p* < 0.001). This improvement was supported by a large effect size, with TSK-11 scores showing a substantial decrease in kinesiophobia (^1^Cohen’s *d* = 1.73, 95% confidence interval [CI] [1.14, 2.32]). All participants tolerated the intervention well, and none required referral to a psychologist.

**TABLE 3:** Fear avoidance levels and Pain levels at baseline and post intervention (*N* = 28).

Outcome measure	Score range	Baseline (Mean ± s.d.)	Post-intervention (Mean ± s.d.)	*p*-value	Cohen’s *d*
TSK-11	11–44	31.3 ± 5.18	17.8 ± 5.86	< 0.001	1.73
BPI Short Form – Severity Subscale	0–10	6.23 ± 2.06	2.69 ± 2.10	< 0.001	1.21
BPI Short Form – Interference Subscale	0–10	5.96 ± 2.00	2.26 ± 2.16	< 0.001	1.25

Note: Statistically and clinically significant decrease seen in all outcomes.

s.d., standard deviation; BPI, Brief Pain Inventory; TSK-11, Tampa Scale of Kinesiophobia-11.

### Pain levels

The results for the BPI-SF are presented as pain severity and pain interference subscales. Table 4 shows a comparison of mean values pre-intervention and post-intervention for the pain interference items. Both subscales were normally distributed at baseline and post-intervention. The results from the parametric paired *t*-test are presented in Table 3, and Figure 2 illustrates the reduction in mean pain severity and pain interference scores at baseline and post intervention. A clinically significant decrease was observed in both pain severity and pain interference, with statistically significant improvements noted for both subscales (*p* < 0.001). These findings were accompanied by large effect sizes with a reduction in pain severity (Cohen’s *d* = 1.21, 95% CI [0.72, 1.70]), and the interference of pain with daily activities (Cohen’s *d* = 1.25, 95% CI [0.76, 1.74]).

**TABLE 4:** Parametric data for the components of the BPI-SF pain interference subscale at baseline and post-intervention (*N* = 28).

Component	Baseline (Mean ± s.d.)	Post-intervention (Mean ± s.d.)	*p*-value
General activity	5.79 ± 2.79	2.43 ± 2.44	< 0.001
Mood	5.82 ± 2.96	1.93 ± 1.82	< 0.001
Walking ability	5.32 ± 2.84	2.29 ± 2.09	< 0.001
Normal work	6.82 ± 2.28	2.93 ± 2.87	< 0.001
Relations with other people	5.39 ± 2.79	2.11 ± 2.47	< 0.001
Sleep	6.89 ± 2.56	2.46 ± 2.49	< 0.001
Enjoyment of life	5.71 ± 2.40	2.32 ± 2.68	< 0.001

Note: Statistically significant decrease seen in all components.

s.d., standard deviation.

## Discussion

Our study demonstrated that a graded exposure therapy programme incorporating PNE was associated with reductions in kinesiophobia in individuals with NSCLBP. The reduction in TSK-11 scores exceeded the minimal clinically important difference, and a large effect size was observed (Cohen’s *d* = 1.73, 95% CI [1.14, 2.32]), suggesting a clinically meaningful decrease in fear of movement. These findings align with earlier Randomised Controlled Trials (RCTs) by Leeuw et al. (2008), Linton et al. (2008), and Woods and Asmundson (2008), which reported reductions in kinesiophobia following graded exposure procedures that typically included a targeted education session and graded exposure to feared activities. Although Linton et al. (2008) did not observe between-group differences for pain or fear outcomes, they noted statistically significant within-group reductions in TSK scores among participants receiving graded exposure. Woods and Asmundson (2008) similarly reported greater reductions in kinesiophobia in the graded exposure group compared with a waitlist control, contributing to a consistent pattern of association across studies. The present study supports this pattern by showing that integrating PNE with graded exposure therapy to feared movements corresponded with a decrease in maladaptive fear responses in this population.

The effectiveness of our study can further be explained through the fear-avoidance model, which outlines how catastrophic interpretations of pain are linked to avoidance, disuse and disability. Pain neuroscience education interrupts this cycle by directly modifying maladaptive beliefs and reframing pain as a protective, rather than damage-based, experience. This cognitive shift may have supported how participants were able to engage with the graded exposure tasks. The results are consistent with the systematic review by Louw et al. (2016), which reported that PNE is often associated with greater reductions in kinesiophobia when delivered alongside other therapeutic modalities, rather than as a stand-alone intervention. In our study, a single session of PNE was included as a complementary component to facilitate participants’ understanding of pain mechanisms and their engagement with graded exposure therapy supporting the therapeutic effect. Building on this, PNE has been associated with changes in threat appraisal and reductions in catastrophising. Neuroimaging evidence further supports this mechanism, showing that reconceptualising pain reduces activation in fear-related regions such as the amygdala and anterior cingulate cortex while enhancing prefrontal inhibitory control (Timmers et al. 2019). This cognitive reframing mechanism has been consistently associated with reductions in fear and maladaptive beliefs (Louw et al. 2016; Moseley 2004). In parallel, graded exposure therapy may help participants gradually unlearn their fear of movement. By repeatedly performing activities they previously avoided – and discovering that these movements were safe – their expectations may have shifted, weakening the learned association between movement and harm (Craske et al. 2014).

In addition to reductions in fear, our study observed significant improvements in pain severity with a large effect size (Cohen’s *d* = 1.21, 95% CI [0.72, 1.70]), consistent with previous studies reporting that graded exposure therapy programmes are associated with lower pain intensity in CLBP populations (Leeuw et al. 2008; Linton et al. 2008; Schemer et al. 2018). Compared with Schemer et al. (2018), the present study showed a similarly large and clinically meaningful reduction, with an average pain decrease of 50%. This improvement may reflect the combined influence of increased movement confidence, reduced pain vigilance, and improved adherence to home programmes supported by structured exercise documentation and education on the importance of consistent practice. Integrating PNE with movement-based therapy has been associated with reduced pain vigilance and awareness while increasing pain pressure thresholds, thereby restoring more adaptive descending inhibitory control (Malfliet et al. 2018). The intervention was also associated with increased self-efficacy, as successfully completing feared tasks appeared to strengthen participants’ confidence in their ability to move safely – an important factor known to be associated with reduced pain interference and disability (George et al. 2010; Linton et al. 2008). Together, these cognitive, behavioural and neurophysiological mechanisms may help explain the improvements in both fear and pain severity observed in our study.

### Strengths and limitations

The strengths of our study included the easy replication of the principles of the graded exposure therapy allowing for better generalisability within different populations, the outcome measures being easy to administer with minimal training and the graded exposure therapy being a cost-effective treatment technique with minimal resources required, making it feasible for both rural and urban settings.

On completion of our study, a few limitations were noted. As our study was only limited to one facility and study site hospital, the sample size was small and therefore not generalisable. Another limitation of the study could be response bias, as blinding of participants was not feasible given the single-group intervention design and reliance on self-reported outcome measures. In addition, the use of self-report outcome measures means that outcomes may reflect participants’ perceptions and expectations rather than purely objective changes. The possibility of residual bias cannot be fully excluded, despite the use of standardised instructions and outcome measures administered by the RA and anonymised data analysis conducted by the PI, who was blinded to pre-test and post-test results and supported by a blinded statistician. The participants’ understanding of the PNE session was not evaluated, as the effects of PNE and activities in the graded exposure therapy protocol were not isolated, making it difficult to determine if participants understood their condition better.

### Implications or recommendations

A recommendation would be for a more recent RCT to be conducted using our study as a baseline in the South African context to assess the true effect of graded exposure therapy using two intervention groups or a control group. Another recommendation would be to stop the graded exposure therapy when the patient has no pain or fear, and possibly add a component of SMART goal setting as recommended by Magalhães et al. (2018) who reported that the progression of the activity would be to increase the intensity according to patients’ functional goals.

## Conclusion

In conclusion, our study evaluated the effect of graded exposure in the management of patients with NSCLBP and kinesiophobia. The findings demonstrate that the graded exposure therapy programme was associated with a large effect size in reducing both pain and kinesiophobia in patients with NSCLBP (*p* < 0.001, Cohen’s *d* = 1.21, 95% CI [0.72, 1.70] and Cohen’s *d* = 1.73, 95% CI [1.14, 2.32]). Future considerations may include the integration of graded exposure therapy with complementary physiotherapy modalities such as PNE, Pilates and cognitive behavioural techniques like progressive muscle relaxation. Additionally, the implementation of an RCT may yield more robust, reliable and generalisable evidence regarding the effect of graded exposure therapy within this clinical population.

## Footnotes

1 For Cohen’s *d*, an effect size of 0.2 = small effect; 0.5 = medium effect; 0.8 = large effect.

## Appendix 1

**TABLE 1-A1:** Pain neuroscience education session teaching and learning plan.

Subject area time allocated	Objectives	Key content	Teaching method	Teaching aids	Evaluation
Ice-breaker and introduction (2 min)	To introduce the topic and give an overview of what will be spoken about	Introduce myself to the participant and have the participant introduce themself and give a fact about who they are. Introduce the topic of study. Purpose of study: ɚ What is chronic pain and kinesiophobia? ɚ How does our body react to a painful stimulus? ɚ What is the fear avoidance model of pain? ɚ How can graded exposure help with the management of chronic pain? ɚ Medical findings of the patient	Lecture and discussion	-	-
Definition of chronic pain and kinesiophobia (3 min)	To determine if participants know what chronic pain and kinesiophobia is	If applicable: Chronic pain is pain in one or more areas that continues for more than 3 months. This pain may cause emotional distress or disability when doing everyday activities (Nicholas et al., 2019). Kinesiophobia is the irrational and excessive fear of movement and activity. Patients have this fear of movement as they think it may cause more pain or re-injury (Vlaeyen et al., 2004).	Lecture and discussion	Poster	Question and answer
Basic neurophysiology of pain (5 min)	To explain the basic neurophysiology of pain	We have sensors all over our bodies that are found at the end of individual neurons. These sensors are specialised and respond to different types of forces and changes. They send information from our tissues to our spinal cord and brain. Neurons in our tissues may respond to different types of stimuli. If those stimuli are dangerous to the tissue, it triggers the neurons to send an alarming signal to the spinal cord and brain. These signals get sent via different pathways and finally to different areas of the brain. When this ‘danger message’ is sent to your brain with other information and messages for processing, the brain’s job is to create a meaningful experience based on all this information. Pain is seen as part of a response to this information according to the area where the signals have ended and involves many parts of the brain. When patients have persistent pain, the tissues have an increased sensitivity due to the constant stimulation and response to the brain. This will result in the information going to the brain no longer reflecting the true health of the tissues, making it seem that there is more danger to the tissues than there actually is. In patients with chronic pain, this system is now sensitive and stimuli that are not related to tissue damage may be perceived by our brain as being dangerous and can be enough to cause pain, without you being aware of it. It is important to note that persistent pain is protective and may not mean that your tissues are getting damaged again. (Butler and Moseley, 2003)	Lecture and discussion	Poster	Question and answer
Basic explanation of the fear avoidance model of pain (5 min)	To explain what the fear avoidance model of pain is	Injury causes a pain experience. This pain may be interpreted as threatening, which causes pain-related fear. This pain-related fear may result in an avoidance of an activity which causes disability or disuse. Disability or disuse can result in you experiencing the pain again If this cycle is not broken, it will increase fear and avoidance and decrease the chance of recovery. (Vlaeyen & Linton, 2000)	Lecture and discussion	Poster and diagram of fear avoidance model of pain	Question and answer
Management of chronic pain using graded exposure at home (5 min)	To educate on the use of graded exposure in the management of chronic lower back pain	Graded exposure is aimed at breaking the fear-avoidance cycle discussed previously and reducing pain levels. It is a process where the patient does an activity at a certain level that was previously feared. Once the patient has a decrease in fear of an activity, the activity will be progressed. This process will continue until the activity is no longer feared. An example will be given and demonstrated.	Lecture and discussion	Poster and demonstration	Question and answer
Questions and answers (5 Min, minutes)	To discuss and/or highlight any areas which were unclear	Participant to bring forward any questions regarding the content discussed and clarity on unclear sections to be given.	Question and answer	-	-

Note: Please see the full reference list of the article, Lord, S., Naidoo, V. & Govind, J., 2026, ‘The effect of graded exposure therapy in patients with non-specific chronic lower back pain and kinesiophobia in Gauteng, South Africa’, *South African Journal of Physiotherapy* 82(1), a2333. https://doi.org/10.4102/sajp.v82i1.2333 for more information.

## Appendix 2

**TABLE 1-A2:** Monitoring tool.

Patient Number:						
Date	Activities	Before activity		After activity		Activity progressed yes or no
		Expectations of fear	Expectations of pain	Level of fear	Level of pain	
						
					
					
					
						
					
					
					
						
					
					
					
						
					
					
					

Numerical rating scale for pain and fear:
